# Changes of trunk muscle stiffness in individuals with low back pain: a systematic review with meta-analysis

**DOI:** 10.1186/s12891-024-07241-3

**Published:** 2024-02-19

**Authors:** Rok Vatovec, Matej Voglar

**Affiliations:** Faculty of Health Sciences, University of Primorska, Polje 42, 6310 Izola, Slovenia

**Keywords:** Muscle properties, neuromuscular control, low back disorders, elastography, myotonometry

## Abstract

**Background:**

Low back pain (LBP) is one of the most common musculoskeletal conditions. People with LBP often display changes of neuromuscular control and trunk mechanical properties, including trunk stiffness. Although a few individual studies have examined back muscle stiffness in individuals with LBP, a synthesis of the evidence appears to be lacking. Therefore, the aim of this systematic review with meta-analysis was to synthesize and evaluate the available literature investigating back muscle stiffness in association with LBP.

**Methods:**

We conducted a systematic review of the literature according to the PRISMA guidelines. We searched Pubmed, Scopus, Web of Science and ScienceDirect for studies, that compared back muscle stiffness, measured either by ultrasound-based elastography or myotonometry, between individuals with and without LBP. Pooled data of the included studies were presented descriptively. Additionally, we performed two meta-analyses to calculate the standardized mean difference between the two groups for resting stiffness of the multifidus and erector spinae muscle. For both meta-analyses, the random effect model was used and the weight of individual studies was calculated using the inverse-variance method. The quality of the included studies was assessed using the Joanna Briggs Institute Critical Appraisal Checklist for Analytical Cross-Sectional studies. Furthermore, the certainty of evidence was evaluated using the GRADE approach.

**Results:**

Nine studies were included in our systematic review. Our results suggest that individuals with LBP have higher stiffness of the multifidus (SMD = 0.48, 95% CI: 0.15 – 0.81, *p* < 0.01; I^2^ = 48 %, *p *= 0.11) and erector spinae at rest (SMD = 0.37, 95% CI: 0.11 – 0.62, *p* < 0.01; I^2^ = 39 %, *p* = 0.14) compared to asymptomatic controls. On the other hand, the evidence regarding muscle stiffness during submaximal contractions is somewhat contradictory.

**Conclusions:**

Based on the findings of this systematic review we conclude that people with LBP may have higher back muscle stiffness compared to asymptomatic controls. Addressing muscle stiffness might represent an important goal of LBP treatment. Nevertheless, our findings should be interpreted with extreme caution due to a limited quality of evidence, small number of included studies and differences in measurement methodology.

**Supplementary Information:**

The online version contains supplementary material available at 10.1186/s12891-024-07241-3.

## Introduction

Low back pain (LBP) is considered one of the most prevalent and debilitating musculoskeletal conditions [1]. It affects the majority of the general population, although prevalence is the highest between the ages of 40 and 60 [2]. LBP frequently results in work absenteeism [3] and associated financial losses [4], which is a major socioeconomic burden. One of the major concerns is the development of chronic LBP and persistent symptoms. Notably, it is estimated that approximately one-third of acute LBP cases develop into chronic LBP [5]. The latter often leads to disability and reduced quality of life [6], thus having a negative impact on the individual’s psychological state [7].

The relatively high prevalence of chronic LBP and persistent symptoms (4.2 – 25.4 %) [8] may be related to changes in structural and neuromuscular properties associated with LBP. In fact, individuals with LBP often display atrophy of paraspinal muscles [9], decreased trunk muscle strength [10], endurance [11], and flexibility [12] as well as alterations in neuromuscular control [13, 14]. Furthermore, several authors have reported higher paraspinal muscle electromyographic (EMG) activity during standing [15] and walking [16]. It has been suggested that the increased muscle activity represents a spine splinting mechanism to increase trunk stiffness and protect the spine from abnormal loading and noxious stimuli [17]. However, Prins and colleagues (2017) [18] concluded in their systematic review that there is no conclusive evidence to support spinal splinting in individuals with LBP.

Despite the conflicting findings of the systematic review by Prins and colleagues (2017) [18], there are some studies reporting increased intrinsic trunk stiffness in individuals with recurrent [17] or experimentally-induced LBP [19]. Even so, measurement of intrinsic trunk stiffness does not indicate, whether potential alterations are related to muscle or connective tissue. Changes of connective tissues have been frequently associated with LBP [20, 21]. While these changes may have an impact on intrinsic trunk stiffness, their contribution has yet to be determined. Importantly, the assessment of back muscle stiffness may represent a window of opportunity to offer additional insights into changes of trunk mechanical properties in association with LBP.

Ultrasound-based measurements and myotonometry are novel approaches that enable the assessment of tissue stiffness by measuring mechanical tissue deformation. Ultrasound-based measurements include shear wave elastography (SWE) and strain elastography (STE). The former measures the velocity of propagation of an ultrasound-induced shear wave within the tissue [22], while the latter measures tissue deformation following mechanical pressure, which is applied manually from the examiner by compressing the skin and underlying tissue [23]. In contrast, myotonometry is performed using myotonometer, a device that applies small compressive forces to the skin and measures deformation of superficial tissues. Both SWE and myotonometry appear to correlate well with exerted muscle force (r = 0.82 – 0.98) [24, 25] and muscle activity (r = 0.70-0.98) [26, 27]. Additionally, both methods were proven to be reliable for measuring back muscle stiffness (SWE: ICC = 0.53 – 0.79, myotonometry: ICC = 0.81 – 0.96) [28, 29].

Current evidence from single studies suggests that people with LBP display higher back muscle stiffness compared to asymptomatic individuals [30–33]. However, due to inconsistent findings and different methodologies used to assess stiffness, a synthesis of the evidence is warranted. Therefore, the aim of our study was to conduct a systematic review and meta-analysis on the changes of resting and active back muscle stiffness in relation to LBP. Our findings could offer additional insights into the relationship between trunk mechanical properties and LBP. We hypothesized that individuals with LBP have higher back muscle stiffness compared to asymptomatic individuals.

## Methods

### Search strategy

A systematic review of the literature was conducted in line with the Preferred Reporting Items for Systematic Reviews and Meta-Analyses 2020 (PRISMA) guidelines [34]. In August 2022 (list of publications extracted on August 1^st^), Pubmed, Scopus, Web of Science and ScienceDirect were screened using the following search term: ("back pain"[Title] OR LBP[Title] OR "back disorder*"[Title] OR "spinal pain"[Title]) AND (myoton*[Title/Abstract] OR stiff*[Title/Abstract] OR elastography[Title/Abstract] OR elastic*[Title/Abstract] OR “mechanical properties”[Title/Abstract]). The search was updated in all databases in October 2023 (list of publications extracted on October 17^th^). The exact search syntaxes for each database are presented in the Supplementary 1. Two authors (RV and MV) independently searched each database for relevant studies. Additionally, reference lists of the included studies were screened for eligible records. The selected studies of both authors were combined. Potential disagreements between authors were resolved upon discussion with an independent third colleague.

### Eligibility criteria

Eligibility criteria were determined using the PICO framework. Cross-sectional/case-control studies that compared stiffness of trunk muscles between adults with and without LBP were included. There were no restrictions based on participants age, duration of symptoms or LBP chronicity (e.g. acute, subacute or chronic). Muscle stiffness had to be assessed via ultrasound-based elastography (SWE or STE) or myotonometry, either during rest or submaximal contraction. Studies were excluded in case of participants with specific spinal pathologies (e.g. spinal stenosis, ankylosing spondylitis or radiculopathy), or absence of a control group. Studies with a within-subject design, comparing the painful and non-painful side, were also excluded.

### Data extraction and data items

The first author (RV) collected relevant data from eligible studies. Data regarding the authors, number, age and sex of participants, measurement methodology, results and main findings were extracted and saved in an Excel sheet (Microsoft Excel, version 2016). Additionally, mean values and standard deviations were extracted from each of the included studies. Authors were contacted by email, to obtain missing data. In case of one study [30], the authors were not able to provide mean and standard deviation values, therefore we adopted their median values as the mean and calculated the standard deviation by dividing the interquartile range with 1.35.

### Assessment of study quality and certainty of evidence

Two authors independently evaluated the quality of the included studies using the Joanna Briggs Institute Critical Appraisal Checklist for Analytical Cross-Sectional studies [35]. This tool consists of eight different items. The interpretation of certain items was slightly modified to match the study design of the included studies. For the third item, we determined, that exposure was measured in a valid and reliable way, if the authors reported the average duration of LBP symptoms. For the fourth item, the authors should have reported the level of pain or disability. Following individual assessments, the authors resolved potential disagreements by consulting a third independent colleague. Inter-rater agreement for individual items was calculated and expressed as the Kappa value. The latter was interpreted as poor (< 0.00), slight (0.00 – 0.20), fair (0.21 –  0.40), moderate (0.41 –  0.60), substantial (0.61 –  0.80) and almost perfect (0.81 –  1.00) [36]. Due to a lack of cut-off values, studies were considered as meeting a minimum of quality, when at least five items were rated “Yes” [37].

The certainty of evidence was evaluated using the GRADE approach [38]. The latter encompasses 5 main domains (risk of bias, imprecision, inconsistency, indirectness and publication bias). The certainty of evidence for each comparison was classified as either high, moderate, low or very low. The grading was modified according to the design of included studies (cross-sectional/case-control). The level of evidence was downgraded if: (a) more than 25 % of studies did not reach the predetermined minimum of quality (risk of bias); (b) a large CI was observed or the total number of participants was < 300 (imprecision); (c) there was a substantial heterogeneity of study results (I^2^ > 50 % or in case of statistical significance (inconsistency); (d) the studies assessed the effects of the disease indirectly (population or outcome not representative of the research question) (indirectness); (e) there was no significant risk for publication bias.

### Quantitative synthesis – meta-analysis of stiffness measures

Pooled data of included studies were presented descriptively. Additionally, we performed two meta-analyses using the Review Manager 5.4 (RevMan 5.4, The Cochrane Collaboration, United Kingdom, 2020). Prior to the analysis, we have confirmed the normality assumption for meta-analysis as described by Wang and Lee (2020) [39]. Furthermore, we performed a preliminary publication bias assessment for each meta-analysis using the Egger’s linear regression test. The first meta-analysis included studies that measured resting multifidus (MF) stiffness using SWE and STE, the other included studies that measured erector spinae (ES) stiffness, either by SWE or myotonometry. We included continuous data and calculated the weight of individual studies using the inverse-variance method. Both analyses were completed by assuming random effects and reported differences between groups as the standardized mean difference (SMD). The effect size was estimated as Cohen’s d and was interpreted as low (d = 0.2), medium (d = 0.5) or large (d = 0.8) [40]. A positive effect size (SMD) value corresponds to higher muscle stiffness in the LBP compared to the control group. The heterogeneity of study results was calculated using I^2^. Due to a small number of included studies, I^2^ was interpreted as “not important” (0 – 30 %), “moderate” (31 – 50 %), “substantial” (51 – 75 %), “considerable” (76 – 100 %) [38].

## Results

### Search results overview

**Fig. 1:**
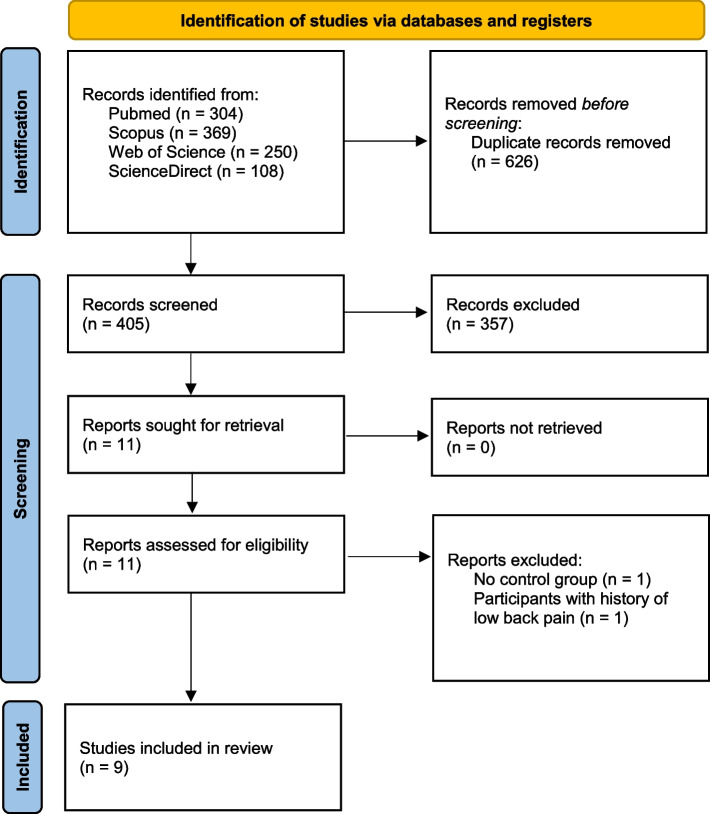
PRISMA flowchart

Overall, our search strategy yielded 1031 results. After duplicate removal, 405 records were screened by title and abstract. Eleven records were sought for retrieval of full publication and subsequently assessed for eligibility. One study was excluded due to absence of a control group [41] and one due to inclusion of participants with history of LBP [42]. Finally, nine studies were included in the systematic review (Fig. 1).

### Qualitative synthesis

Nine studies were included in the systematic review. Altogether, 639 participants were included, 315 with and 324 without LBP. Overall, the mean age of the included participants was 39.1 years. More specifically, the mean age of participants with LBP was 40.8 years, while the mean age of participants from the control group was 37.4 years. One study included only the elderly [33], whereas the others included adults in general. One study included only male participants [43]. Six studies included only participants with chronic LBP, which was defined either as consecutive pain for more than 3 months or non-continuous pain for greater than 6 months with pain on at least half of the days [30, 32, 33, 43–45]. Alcaraz-Clariana et al., 2021 included participants with acute [46] and subacute [47] LBP in their two studies. On the other hand, Koppenhaver et al. [31] did not recruit participants based on the duration of LBP. Characteristics of included studies, that measured stiffness using elastography and myotonometry are presented in Tables 1 and 2, respectively.

**Table 1:** Characteristics of included studies, that measured stiffness with elastography

**Authors, year,** **country**	**Participants, sex, age**	**LBP subtype**	**Measurement method**	**Results**		**Main findings**
				LBP	CON	
Chan et al., 2012 [43], China	*n* = 24 (all M) 12 LBP: 36.6 ± 2.9 y 12 CON: 25.2 ± 1.1 y	Chronic LBP	STE (MF – L4) [kPa]: Resting Standing Forward stoop 25° Forward stoop 45°	40.2 ± 5.0 67.2 ± 9.3* 101.2 ± 9.4* 127.1 ± 9.8*	36.9 ± 4.8 54.2 ± 7.0 93.9 ± 14.0 108.1 ± 17.2	Individuals with LBP have higher MF stiffness in standing and stooping. No differences were observed in resting stiffness.
Masaki et al., 2017 [32], Japan	*n* = 32 9 LBP (1 M, 8 F): 44.7 ± 13.0 y 23 CON (8 M, 15 F): 34.7 ± 10.2 y	Chronic LBP	SWE [kPa]: ES: 7 cm lateral to L3 MF: lateral to L4	3.7 ± 1.1 5.6 ± 1.1*	3.5 ± 1.1 4.8 ± 0.8	Individuals with LBP have higher MF resting stiffness. No differences were observed for ES stiffness.
Murillo et al., 2019 [44], United Kingdom	*n* = 30 15 LBP (7 M, 8 F): 29.4 ± 10.8 y 15 CON (8 M, 7 F): 26.7 ± 5.4 y	Chronic LBP	SWE [kPa]: SMF: L3 under TLF DMF: L3 over laminae A-SMF	10.2 ± 4.2* 14.4 ± 2.6 N/A	6.8 ± 1.7 15.4 ± 2.8 N/A	Individuals with LBP have higher resting stiffness of SMF, but not DMF. No differences were observed for SMF stiffness during contraction.
Koppenhaver et al., 2020 [31], USA	*n* = 120 60 LBP (36 M, 24 F): 32.2 ± 7.3 y 60 CON (26 M, 34 F): 31.0 ± 8.0 y	Not defined	SWE [kPa]: ES: symptomatic level MF: symptomatic level or L4/L5 facet joint A-MF	6.4 ± 3.4* 6.8 ± 3.2* 20.8 ± 10.8	4.5 ± 1.7 5.7 ± 2.0 22.6 ± 9.8	Individuals with LBP have higher resting ES and MF stiffness. No differences were observed for MF stiffness during contraction.
Pinto et al., 2022 [45], China	*n* = 151 78 LBP (32 M, 46 F): 46.0 y 73 CON (26 M, 52 F): 48.0 y	Chronic LBP	SWE [kPa]: MF: facet L4/L5 MF: facet L5/S1	43.3 ± 21.5 43.5 ± 21.2	41.3 ± 18.7 41.9 ± 19.4	No differences were observed for resting SMF stiffness.

*Legend*: *M* Male, *F* Female, [ ] unit of measurement, *LBP* Low back pain, *CON* Control, *MF* M. multifidus, *SMF* Superficial multifidus, *DMF* Deep multifidus, *ES* m. erector spinae, *SWE* Shear wave elastography, *STE* Strain elastography, *TLF* Thoracolumbar fascia, *A* Active stiffness, * = *p* < 0.05

**Table 2:** Characteristics of included studies, that measured stiffness with myotonometry

**Authors, year,** **country**	**Participants, sex, age**	**LBP subtype**	**Measurement method**	**Results**		**Main findings**
				LBP	CON	
Wu et al., 2019 [33], China	*n* = 80 40 LBP (20 M, 20 F): 63.4 ± 8.4 y 40 CON (20 M, 20 F): 63.6 ± 6.9 y	Chronic LBP	MyotonPRO: Stiffness [N/m]	319.2 ± 73.8*	277.1 ± 44.7	Individuals with LBP have higher stiffness.
Ilahi et al., 2020 [30], USA	*n* = 50 25 LBP (9 M, 16 F): 33.1 ± 1.4 y 25 CON (9 M, 16 F): 31.4 ± 1.5 y	Chronic LBP	MyotonPRO: Stiffness [N/m]	239.2 ± 58.6	237.3 ± 89.2	No differences in stiffness between individuals with and without LBP. Females with LBP have higher stiffness.
Alcaraz-Clariana et al., 2021b [47], Spain	*n* = 86 43 LBP (28 M, 15 F): 40.2 ± 12.3 y 43 CON (28 M, 15 F): 39.2 ± 11.3 y	Subacute LBP	MyotonPRO: Stiffness [N/m]	303.8 ± 64.8	284.2 ± 82.6	No differences in stiffness between individuals with and without LBP.
Alcaraz-Clariana et al., 2021a [46], Spain	*n* = 66 33 LBP (22 M, 11 F): 41.9 ± 14.8 y 33 CON (20 M, 13 F): 37.0 ± 10.9	Acute LBP	MyotonPRO: Stiffness [N/m]	289.9 ± 76.2	283.7 ± 75.4	No differences in stiffness between individuals with and without LBP.

*Legend*: *M* Male, *F* Female, [ ] unit of measurement, *LBP* Low back pain, *CON* control, * = *p* < 0.05

Four of the included studies used SWE to assess muscle stiffness [31, 32, 44, 45], one study used STE [43]. All five studies measured resting lumbar MF muscle stiffness. The probe location was either lateral to the L4-L5 interspace [32, 43, 45], L3 level [44] or at the most symptomatic level [31]. The only study that used STE and expressed muscle stiffness as the Young modulus found no difference between LBP patients and the control group [43]. Conversely, three studies that measured the shear modulus using SWE reported significantly higher resting lumbar MF stiffness in participants with LBP [31, 32, 44]. One study found no difference in resting lumbar MF shear modulus between participants with and without LBP [45]. Two of the included studies evaluated stiffness of the MF during submaximal contraction, which was achieved by performing a prone arm lift [31] or isometric trunk extension [44]. Both studies found no differences between the two groups. One study showed higher lumbar MF Young modulus during standing and forward stooping [43]. Two studies included resting ES shear modulus as an outcome measure. One found no difference between groups [32], whereas the other showed higher ES resting stiffness in individuals with LBP [31].

Four of the included studies evaluated ES myofascial stiffness using myotonometry. One study found higher stiffness in older participants with chronic LBP [33], while one reported higher stiffness only when comparing female groups [30]. Two studies did not report any differences in stiffness in individuals with acute [46] and subacute LBP [47].

### Methodological quality of studies

The results of study quality assessment are presented in Table 3. Overall, the inter-rater agreement was high (97 %). Kappa values for individual items ranged from 0.73 – 1.0, indicating a substantial to almost perfect strength of agreement. For items “clearly defined inclusion criteria”, “detailed subject description”, “outcomes measured in a valid and reliable way” and “appropriate statistical analysis use” all studies met the criteria. Item “valid and reliable measurement of exposure” was met by 38 % [31–33], “objective criteria for measurement of condition” by 88 % [31–33, 43–47], “identified confounding factors” by 38 % [31, 43, 45] and “strategies to deal with confounding factors” by 25 % [31, 45] of the included studies. One study did not achieve the predetermined minimum of quality (at least five “Yes”).

**Table 3:** Study quality assessment using the Joanna Briggs Institute Critical Appraisal Checklist for Analytical Cross-Sectional studies

**Authors**	**1**	**2**	**3**	**4**	**5**	**6**	**7**	**8**	**Total score**
Chan et al. 2012 [43]	1	1	0	1	1	0	1	1	6/8
Masaki et al. 2017 [32]	1	1	1	1	0	0	1	1	6/8
Murillo et al. 2019 [44]	1	1	0	1	0	0	1	1	5/8
Koppenhaver et al. 2020 [31]	1	1	1	1	1	1	1	1	8/8
Pinto et al. 2022 [45]	1	1	0	1	1	1	1	1	7/8
Wu et al. 2019 [33]	1	1	1	1	0	0	1	1	6/8
Ilahi et al. 2020 [30]	1	1	0	0	0	0	1	1	4/8
Alcaraz-Clariana et al. 2021a [46]	1	1	0	1	0	0	1	1	5/8
Alcaraz-Clariana et al. 2021b [47]	1	1	0	1	0	0	1	1	5/8
Inter-rater agreement (Kappa)	1.0	1.0	0.73	1.0	0.77	1.0	1.0	1.0	

*Legend*: 1: clearly defined inclusion criteria, 2: detailed subject description, 3: valid and reliable measurement of exposure, 4: objective criteria for measurement of condition, 5: identified confounding factors, 6. strategies to deal with confounding factors, 7. outcomes measured in a valid and reliable way, 8. appropriate statistical analysis use; score 1: Yes, score 0: No

### Differences between LBP patients and controls – Meta-analyses

**Fig. 2:**
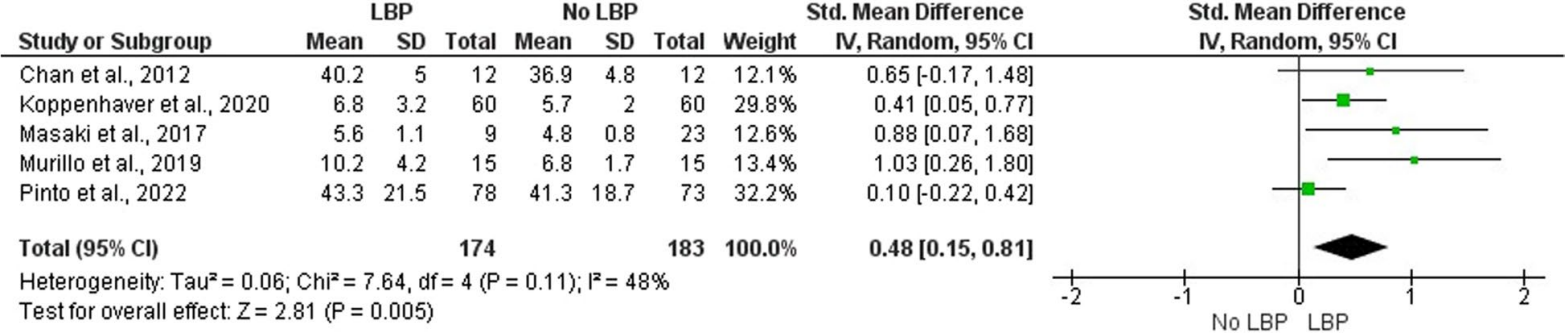
Meta-analysis for multifidus stiffness in people with and without low back pain Legend: LBP low back pain, SD standard deviation

The results of our meta-analysis (Fig. 2) revealed significantly higher resting MF stiffness in participants with LBP compared to controls with a small effect size (SMD = 0.48, 95% CI: 0.15 – 0.81, *p* < 0.01). The heterogeneity of study results was moderate, albeit not significant (I^2^ = 48 %, 95% CI: 0 – 94 % *p* = 0.11). The results of Egger’s test indicate a significant risk for publication bias (*p* = 0.04) (Supplementary 2). The certainty of evidence was low.

**Fig. 3:**
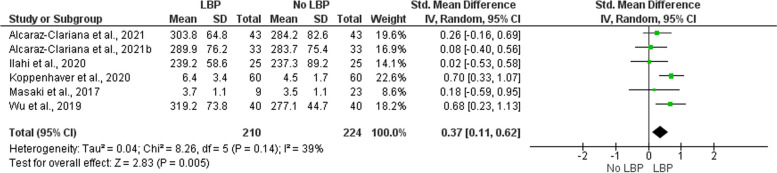
Meta-analysis for erector spinae stiffness in people with and without low back pain Legend: LBP low back pain, SD standard deviation

Likewise, our meta-analysis (Fig. 3) showed significantly higher resting ES muscle stiffness in participants with LBP compared to controls with a small effect size (SMD = 0.37, 95% CI: 0.11 – 0.62, *p* < 0.01). The heterogeneity of study results was moderate, albeit not significant (I^2^ = 39 %, 95% CI: 10 – 82 %, *p* = 0.14). The results of Egger’s test were not significant (*p* = 0.21) (Supplementary 2). The certainty of evidence was moderate.

## Discussion

The aim of this systematic review with meta-analysis was to evaluate changes in muscle stiffness in individuals with LBP. Our meta-analyses revealed that resting MF and ES stiffness is significantly higher in individuals with LBP. However, the certainty of evidence was low to moderate at best, therefore our findings should be interpreted with extreme caution. In contrast, the results of studies, that measured muscle stiffness during submaximal contractions appear, to be somewhat conflicting.

Our findings indicate that in general LBP may be associated with increased resting trunk muscle stiffness. This is in some contrast with an extensive systematic review conducted by Prins et al. (2017) [18], which found no differences in trunk stiffness between individuals with and without LBP. The authors included studies that measured trunk stiffness and muscle activity following unexpected perturbations. Intrinsic trunk stiffness depends on both muscle and connective tissue stiffness. That being said, it is possible that the observed increased trunk extensor stiffness represents a compensation for altered connective tissue stiffness and therefore does not result in increased intrinsic trunk stiffness. However, since LBP is associated with increased rather than decreased connective tissue stiffness [21, 48], this should manifest as higher intrinsic trunk stiffness. Several studies included in the aforementioned review applied perturbations to the upper or lower extremities, thus including another potential source of compensatory strategies that could influence trunk kinematics (e.g. elbow or hip movements). Also, the majority of studies measured EMG activity as the primary outcome, which does not per se reflect trunk mechanical properties.

Muscle stiffness appears to correlate well with muscle force [24] and EMG activity [27]. The observed higher levels of trunk muscle stiffness in individuals with LBP could be associated with increased EMG muscle activity. Indeed, individuals with LBP often show increased trunk muscle activity during standing [15], walking [16] or forward flexion [49]. Nevertheless, in a recent review, Van Dieën et al. (2019) [50] concluded that changes in muscle activity and motor control in individuals with LBP display high intra- and interindividual variability. In fact, while some studies reported increased trunk muscle activity during standing [51], others found either no differences [52] or even lower activity levels [53]. The authors added that the heterogeneous nature of LBP should be taken into account when studying associations between motor control and LBP. In addition to muscle activity, the stiffness of a muscle is also determined by its structural and morphological characteristics. Changes in MF structure and morphology, such as atrophy and fat infiltration, have been previously described in individuals with LBP [54]. However, these changes are in odds with our results, as both atrophy [55, 56] and fat infiltration [57] are associated with decreased muscle stiffness. Our findings could be explained by other morphological changes. For instance, experimentally induced intervertebral disc degeneration in animal specimens seems to lead to a more pronounced increase in muscle bundle compared to muscle fibre stiffness, which is most likely associated with proliferation of connective tissue [58]. In terms of fibre distribution, LBP can lead to a transition from type I to type II muscle fibres of the MF [59]. Type II fibres have been shown to be less stiff compared to type I [60], hence a reduction in muscle stiffness would be expected. However, since type II fibres are less fatigue-resistant, it is possible that muscles may exhibit higher levels of fatigue throughout daily activities. Kumamoto and colleagues (2021) [61] found that a 60-s bout of sustained trunk extension resulted in increased MF stiffness. Accordingly, fatiguing of type II fibres in individuals with LBP might lead to increased muscle stiffness. Conversely, study findings from other muscle groups suggest that resting muscle stiffness either remains unchanged [62] or decreases [63] following fatiguing protocols, hence definitive conclusions cannot be drawn. In summary, although the exact underlying mechanism of increased trunk muscle stiffness in LBP is yet to be proven, it is clear that several factors may play an important role.

Although our meta-analysis revealed a significant difference in resting MF stiffness between participants with and without LBP, two studies found no differences between groups [43, 45]. Chan and colleagues (2012) [43] assessed stiffness using the STE, which seems to be more examiner-dependent compared to SWE [64]. Pinto et al. (2022) [45] on the other hand utilized SWE and obtained substantially higher (43.3 kPa) values of resting MF stiffness compared to the other included studies (4.8 – 6.8 kPa). Conversely, with the exception of Wu et al. (2019) [33], all studies that measured trunk extensor stiffness using myotonometry found no differences between participants with and without LBP. Among these, two included only participants with acute [46] and subacute LBP [47]. Thus, it is plausible that changes in muscle stiffness could be specific to chronic LBP. Yet, Ilahi et al. (2020) [30] included participants with chronic LBP and found no differences when analyzing both genders, although a significant difference was observed when only female subjects were compared. Importantly, we must consider the limitations of myotonometry, when interpreting the findings of the aforementioned studies. Myotonometry measures the mechanical deformation of superficial tissues following the application of a compressive force. Therefore, tissues other than muscle, such as superficial connective tissue and subcutaneous fat may also affect the results. Not surprisingly, Bravo-Sanchez et al. (2021) [30] reported a positive correlation between thigh superficial connective tissue thickness, determined by magnetic resonance imaging, and muscle stiffness, measured with myotonometry. Individuals with LBP were shown to have a higher cross-sectional area of the superficial thoracolumbar fascia [21], thus it is plausible that increased stiffness might be partially related to changes of the connective tissue and not by the changes in the muscle itself.

The evidence regarding changes in MF stiffness during submaximal contraction in people with LBP is somewhat conflicting. Two of the included studies found no differences in active MF stiffness [31, 44]. Murillo et al. [44] measured muscle stiffness during an isometric trunk extension test, whereas Koppenhaver et al., 2020 [31] used the prone contralateral arm lift test. Although this test has been shown to elicit contraction and increased activity of the MF [65], one must be cautious when interpreting their results. Indeed, during this task intermuscular coordination and muscle activity could vary considerably between individuals. This is partially supported by a relatively high standard deviation compared to the mean (22.6 ± 9.8) reported by the authors. In contrast to the previously mentioned studies, Chan et al. 2012 [43] observed increased MF stiffness in individuals with LBP during static forward stooping (25 and 45° spinal flexion). Although we cannot conclusively determine the origin of this finding, we offer some possible explanations. During forward stooping the spine assumes a forward flexed position. In this position the forces acting on the spine differ from loading close to a neutral position in standing. More specifically, the spine is subjected to shear forces, leading to an increased need for stability [66]. Consequently, the central nervous system increases trunk muscle coactivation to meet stability demands, resulting in increased muscle stiffness. In individuals with LBP this increase in trunk bracing could be more pronounced due to impaired motor control [50], which would explain higher levels of muscle stiffness. Furthermore, individuals with LBP often adopt the belief that their spine needs to be protected during bending [67]. Increased muscle activity and stiffness might represent a strategy to deal with this belief. To the best of our knowledge, there are currently no studies using myotonometry for assessment of muscle stiffness during submaximal contraction in people with LBP.

A higher degree of muscle stiffness might be an important factor in the occurrence or persistence of LBP symptoms and disability. In an acute episode of LBP elevated trunk muscle stiffness could limit excessive spinal movement, thus likely protecting the spine from harmful loads and further injury [17]. Conversely, long-term increases in muscle stiffness could result in increased compressive forces on the spine [68], possibly leading to facet joint or intervertebral disc impairments. Also, increased muscle stiffness could lead to decreased movement efficiency and an associated higher energy expenditure. All things considered, reduction of muscle stiffness could be one of the goals when treating LBP patients. Interestingly, research has shown that some of the frequently applied physiotherapeutic interventions in LBP treatment such as dry needling [69], sustained natural apophyseal glides [70], electrotherapy and myofascial release [71] lead to a reduction of muscle stiffness. While it is not clear whether this reduction in stiffness is mediated by improvement in pain or vice versa, it does indicate a potential role of addressing muscle stiffness in LBP management. In terms of examination, muscle stiffness assessment might be useful to evaluate progress of patients with LBP in clinical settings. In this context, myotonometry might be a more suitable option due to its practical applicability.

### Limitations

Finally, some limitations of our review should be noted. First, we included only case-control studies, therefore based on the findings of included studies we cannot infer on a cause-effect relationship. In this regard, future prospective studies are warranted. Second, for our first meta-analysis on MF stiffness we found a significant risk for publication bias, as calculated by the Egger’s regression test. Notably, the studies with the highest effect sizes included smaller samples and had the highest 95% CI intervals. Therefore, we cannot exclude the possibility that smaller studies with contradictory findings have not been published. With regards to our second meta-analysis we included studies which supposedly measured ES stiffness, regardless of the used method. Although myotonometry does measure ES stiffness to some extent, other tissues such as thoracolumbar fascia and subcutaneous fat, may influence the results. Furthermore, we observed moderate heterogeneity with very large 95% CI for both comparisons, thus we emphasize the importance of extreme caution when interpreting the results of our study. Another limitation is the lack of comparison between males and females. Moreover, on average the included studies achieved the predetermined minimum of quality, although the majority did not consider possible confounding factors, that could have influenced their results. We strongly recommend future studies to consider possible confounding factors and adopt appropriate strategies to deal with them. Lastly, although a comprehensive search of the literature was conducted, there remains grey literature, such as unpublished studies and conference papers, that was not considered in the search.

## Conclusions

The aim of this systematic review with meta-analysis was to investigate changes in trunk muscle stiffness in relation to LBP. Our findings suggest that there is low to moderate certainty of evidence of higher resting MF and ES stiffness in individuals with LBP compared with asymptomatic controls. On the other hand, evidence for altered MF stiffness during submaximal contraction is somewhat conflicting. All things considered, we advise extreme caution when interpreting our study results, due to a limited certainty of evidence, small number of included studies and differences in methodology of tissue stiffness assessment.

### Practical implications

Increased back muscle stiffness could play a role in the persistence of symptoms and could represent a potential treatment goal in LBP management. Additionally, muscle stiffness may be used as an outcome measure for the evaluation of progress in patients with LBP.

### Supplementary Information

**Additonal file 1.**

**Additional file 2.**

## Data Availability

The datasets used and/or analysed during the current study are available from the corresponding author on reasonable request.

## Abbrevations

LBP: Low back pain
EMG: Electromyography
SWE: Shear-wave elastography
STE: Strain elastography
MF: m. multifidus
ES: m. erector spinae
SMD: Standardized mean difference
