# Supplementary material for: Changes of trunk muscle stiffness in individuals with low back pain: a systematic review with meta-analysis
Source: BMC Musculoskelet Disord. 2024 Feb 19;25:155. doi: 10.1186/s12891-024-07241-3 (PMC10875766; doi:10.1186/s12891-024-07241-3)
Supplement: Supplementary file 1 — Additonal file 1. [file 12891_2024_7241_MOESM1_ESM.docx]

**Pubmed**

("back pain"[Title] OR LBP[Title] OR "back disorder*"[Title] OR "spinal pain"[Title]) AND (myoton*[Title/Abstract] OR stiff*[Title/Abstract] OR elastography[Title/Abstract] OR elastic*[Title/Abstract] OR "mechanical properties"[Title/Abstract])

**Scopus**

TITLE ( "back pain" OR lbp OR "back disorder*" OR "spinal pain" ) AND TITLE-ABS ( myoton* OR stiff* OR elastography OR elastic* OR "mechanical properties" )

**WoS**

TI=("back pain" OR LBP OR "back disorder*" OR "spinal pain") AND AB=(myoton* OR stiff* OR elastography OR elastic* OR "mechanical properties")

**ScienceDirect**

Title, abstract, keywords: myoton OR stiff OR elastography OR elastic OR "mechanical properties"

Title: "back pain" OR LBP OR "back disorders" OR "spinal pain"
