# Supplementary figures and images for: Changes of trunk muscle stiffness in individuals with low back pain: a systematic review with meta-analysis
Source: BMC Musculoskelet Disord. 2024 Feb 19;25:155. doi: 10.1186/s12891-024-07241-3 (PMC10875766; doi:10.1186/s12891-024-07241-3)

Funnel plot for multifidus stiffness


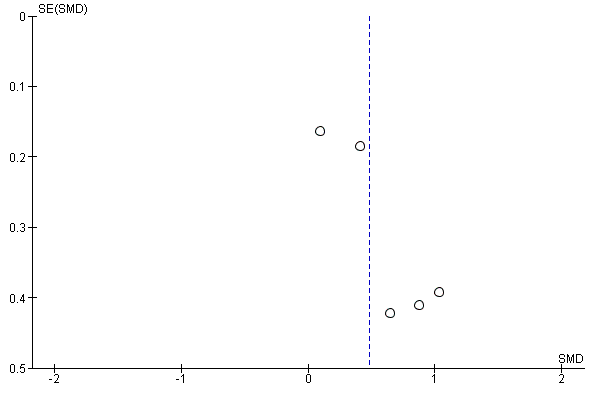


Funnel plot for erector spinae stiffness


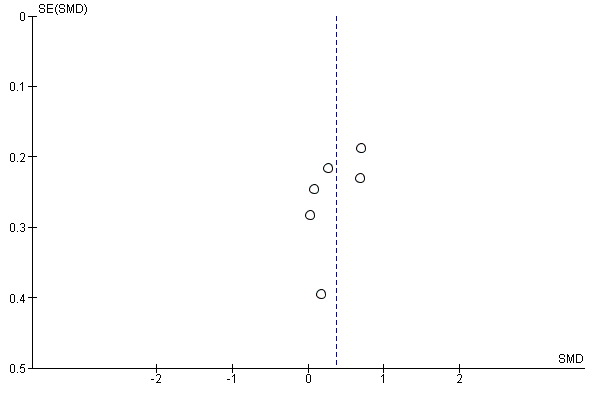

Supplement: Supplementary file 2 — Additional file 2. [file 12891_2024_7241_MOESM2_ESM.docx]
